# Exact Solutions for Non-Isothermal Flows of Second Grade Fluid between Parallel Plates

**DOI:** 10.3390/nano13081409

**Published:** 2023-04-19

**Authors:** Evgenii S. Baranovskii

**Affiliations:** Department of Applied Mathematics, Informatics and Mechanics, Voronezh State University, 394018 Voronezh, Russia; esbaranovskii@gmail.com

**Keywords:** non-Newtonian fluids, second grade fluids, nanofluids, Poiseuille flow, heat and mass transfer, no-slip condition, Navier slip condition, threshold slip condition, exact solutions

## Abstract

In this paper, we obtain new exact solutions for the unidirectional non-isothermal flow of a second grade fluid in a plane channel with impermeable solid walls, taking into account the fluid energy dissipation (mechanical-to-thermal energy conversion) in the heat transfer equation. It is assumed that the flow is time-independent and driven by the pressure gradient. On the channel walls, various boundary conditions are stated. Namely, we consider the no-slip conditions, the threshold slip conditions, which include Navier’s slip condition (free slip) as a limit case, as well as mixed boundary conditions, assuming that the upper and lower walls of the channel differ in their physical properties. The dependence of solutions on the boundary conditions is discussed in some detail. Moreover, we establish explicit relationships for the model parameters that guarantee the slip (or no-slip) regime on the boundaries.

## 1. Introduction

Many real fluids and fluid-like materials used in nanotechnologies belong to the class of *fluids of complexity N* (see [1,2]). For these fluids, the Cauchy stress tensor T is given by the relation

$$T=-pI+F(A_{1},\ldots ,A_{N}),$$

where

- *p* is the pressure;
- I is the identity tensor;
- F is a frame indifferent response function;
- $A_{1},\ldots ,A_{N}$ are the first *N* Rivlin–Ericksen tensors:
  $$\begin{array}{cc} A_{1} & \overset{\mathrm{def}}{=}\nabla v+{\left(\nabla v\right)}^{⊤},\\ A_{j} & \overset{\mathrm{def}}{=}\frac{d}{dt}A_{j-1}+A_{j-1}\nabla v+{\left(\nabla v\right)}^{⊤}A_{j-1},\;\;\;j=2,\ldots ,N; \end{array}$$
- v is the velocity field;
- ∇v denotes the velocity gradient;
- ${\left(\nabla v\right)}^{⊤}$ denotes the transpose of the velocity gradient;
- the differential operator d/dt is the material time derivative,
  $$\frac{d}{dt}A_{j-1}\overset{\mathrm{def}}{=}\frac{\partial }{\partial t}A_{j-1}+\left(v\cdot \nabla \right)A_{j-1}.$$

If F is a polynomial of degree *N*, then the corresponding fluid is called a *fluid of grade N*.

An incompressible Newtonian fluid

$$T=-pI+\mu A_{1},\;\mu >0,$$

is a fluid of grade 1. Fluids with shear-dependent viscosity, for which the constitutive equation is given by the equality

$$T=-pI+\mu \left(A_{1}\right)A_{1},$$

belong to the class of fluids of complexity 1.

In the present paper, we deal with the second grade fluids:

(1) $$T=-pI+\mu A_{1}+\alpha_{1}A_{2}+\alpha_{2}A_{1}^{2},$$

where μ is the viscosity coefficient, μ>0, while $\alpha_{1}$ and $\alpha_{2}$ are the normal stress moduli.

Note that if the equality $\alpha_{1}+\alpha_{2}=0$ holds, then one can rewrite (1) as follows:

(2) $$T=-pI+\mu A_{1}+\alpha_{1}\frac{d}{dt}A_{1}+\alpha_{1}\left(A_{1}W-WA_{1}\right),$$

where W is the vorticity tensor defined by

$$W\overset{\mathrm{def}}{=}\frac{1}{2}\left(\nabla v-{\left(\nabla v\right)}^{⊤}\right).$$

The nonlinear constitutive relations (1) and (2), as well as their various modifications, are often used in the dynamics modeling of nanoscale fluids (see, for example [3,4,5,6]).

Many dilute polymer solutions belong to the class of nanofluids that obey (1). It is well known that the addition of a small amount of polymer nanoparticles to water almost does not change the physical characteristics of the solution, such as the density and the viscosity, but the fluid gains some relaxation properties. An important consequence is that the friction drag drastically decreases for both internal problems (flow in pipes) and external problems (flow past bodies). This effect was discovered by Toms [7] and stimulated in a series of experimental and theoretical studies of the dynamics of aqueous solutions of polymers (see [8,9,10,11,12,13,14,15] and the references therein).

A model for the motion of polymer solutions, considering their relaxation properties, was proposed by Voitkunskii, Amfilokhiev, and Pavlovskii [16]. Using ideas of the hereditary theory of viscoelasticity [2,17], these authors introduced the Maxwell-type relationship between the Cauchy stress tensor T and the deformation rate tensor D:

(3) $$T=-pI+2\mu D\left(v\left(\cdot ,t\right)\right)+\frac{2\chi }{\tau }\int_{-\infty }^{t}\exp\left(-\frac{t-s}{\tau }\right)\frac{d}{ds}D\left(v\left(\cdot ,s\right)\right)\;ds,$$

where

- $D\left(v\right)\overset{\mathrm{def}}{=}\left(\nabla v+{\left(\nabla v\right)}^{⊤}\right)/2$;
- τ is the shear stress relaxation time, τ>0;
- χ is the relaxation viscosity coefficient, χ>0.

Using the smallness of the parameter τ, Pavlovskii [18] performed the asymptotic expansion of the integral term from (3) with respect to $\tau \to 0^{+}$. Retaining only the first term of this expansion, he obtained

(4) $$T=-pI+2\mu D+2\chi \frac{d}{dt}D\left(v\right).$$

Clearly, the last relation is a simplified version of (2) with $\alpha_{1}=\chi$ under the assumption that the product $\alpha_{1}\left(A_{1}W-WA_{1}\right)$ is small compared to the other terms in equality (2) and can be dropped.

The adequacy of the rheological model (4) has been supported by experimental studies. In particular, (4) is considered as a suitable constitutive relationship for low-concentrated aqueous solutions of polyethylenoxide, polyacrylamide, and guar gum [10,11]. The analysis of exact solutions of the corresponding nonlinear motion equations confirms that polymer nanoparticles added to water even in small amounts have a significant influence on the flow pattern [19].

A fluid modeled by (1) is compatible with the thermodynamic laws and stability principles if the following restrictions are imposed on the material constants $\alpha_{1}$ and $\alpha_{2}$:

(5) $$\alpha_{1}\geq 0,\;\alpha_{1}+\alpha_{2}=0,$$

for details, see [20]. Moreover, Fosdick and Rajagopal [21] showed that for arbitrary values of the sum $\alpha_{1}+\alpha_{2}$, with $\alpha_{1}<0$, a fluid totally filling a bounded domain and adhering to the boundary of this domain exhibits an anomalous behavior not expected with real fluids. For a detailed discussion on the physical background, we refer readers to the critical and extensive historical review of second grade (and higher-order) fluid models [22].

Assuming that (5) holds, we introduce the notation $\alpha =\alpha_{1}=-\alpha_{2}$ and rewrite (1) as follows:

(6) $$T=-pI+\mu A_{1}+\alpha A_{2}-\alpha A_{1}^{2}.$$

The aim of the present paper is to obtain exact solutions for non-isothermal steady-state flows of the fluid (6) in a flat infinite channel with impermeable solid walls.

The pointed feature of our work is that different types of boundary conditions on channel walls are used. In addition to the standard no-slip boundary condition v=0, we will consider the threshold slip conditions, which include Navier’s slip conditions as a limit case, as well as mixed boundary conditions, which are suitable for the case when the upper and lower walls have different physical properties. Importance of the wall slip effect and its influence on various characteristics of fluid flows, especially in the case of non-Newtonian fluids, are mentioned in many studies (see [23,24,25,26,27] and the references therein). In particular, as noted in [27], the study of wall slip is very important because it can be used to determine the true rheology of complex fluids by correcting data for slipping effects and explaining a mismatch of rheological data that are obtained from rheometers having different geometries.

Another feature of this paper is that we take into account the fluid energy dissipation (mechanical-to-thermal energy conversion) in the heat transfer equation. In many studies (see, e.g., [28,29]), the influence of the Rayleigh dissipation function is neglected because the mathematical analysis of heat and motion equations are considerably simplified due to artificially vanishing the term involving a quadratic function of space derivatives of the velocity field. However, from the physical point of view, it is more interesting not to use this simplifying assumption and keep all nonlinearities in the original equations [30,31,32].

For each boundary value problem under consideration, we construct exact solutions which determine the velocity field, the temperature, and the pressure in the flow region. Since the used boundary conditions allow for various types of “fluid–solid walls” interactions, we establish explicit relationships for model parameters that guarantee the slip/no-slip regime on the channel walls. Note that the obtained results are valid and new for a Newtonian fluid too, which can be considered as the limit case of a second grade fluid as $\alpha \to 0^{+}$.

The present paper is a continuation of [33,34], in which analogous boundary value problems were considered for isothermal flows. It should be mentioned that many exact solutions for steady and time-dependent motions of the second grade fluids have been established by different authors. The first exact solutions for unsteady flows of this class of non-Newtonian fluids seem to be those of Ting [35], both in rectangular and cylindrical domains. In particular, he showed that solutions are unbounded when $\alpha_{1}<0$. Ting’s results were extended by Coleman et al. [36], who performed a mathematical analysis (instability, uniqueness, and nonexistence theorems) of initial boundary value problems describing non-steady simple shearing flows of second grade fluids provided that $\alpha_{1}<0$. Hron et al. [37] investigated exact solutions for steady-state flows of fluids of complexity 2 in a plane channel and a cylindrical pipe and flows between two rotating concentric cylinders subject to Navier’s slip boundary condition. Exact solutions for the velocity field corresponding to the second problem of Stokes were obtained in [38] by the Laplace transform method. Fetecau et al. [39] analytically studied the magnetohydrodynamic (MHD) flow of second grade fluids with Caputo–Fabrizio time fractional derivatives over a moving infinite flat plate. In [40], it was shown that the governing equations for the fluid velocity and non-trivial shear stress corresponding to some isothermal MHD unidirectional motions of second grade fluids through a porous medium have identical forms. Fetecau and Vieru [41] provided the first exact general solutions for isothermal MHD flows of incompressible second grade fluids between infinite horizontal parallel plates embedded in a porous medium. Note also that there are numerous mathematical studies concerning the existence and uniqueness of solutions to the motion equations of second grade fluids [42,43,44,45,46] as well as optimal control flow problems [47,48,49,50]. The literature on these fluids continues to grow, providing a deeper understanding of the physical processes and support for modern technological advances, in particular for nanotechnologies.

## 2. Statements of Boundary Value Problems

The non-isothermal steady flow of a fluid with constant density is governed by the following system of equations:

(7) $$\begin{array}{c} \rho (v\cdot \nabla )v=\mathrm{div}T+\rho g, \end{array}$$

(8) $$\begin{array}{c} \nabla \cdot v=0, \end{array}$$

(9) $$\begin{array}{c} \rho \left(v\cdot \nabla \right)\theta -\kappa \nabla^{2}\theta =\omega +\Phi , \end{array}$$

where

- ρ is the fluid density, ρ>0;
- $v={\left(v_{1},v_{2},v_{3}\right)}^{⊤}$ is the velocity vector;
- T is the Cauchy stress tensor;
- $g={\left(g_{1},g_{2},g_{3}\right)}^{⊤}$ is the external force per unit mass;
- θ is the temperature;
- κ is the thermal conductivity, κ>0;
- ω is the heat source intensity;
- Φ is the Rayleigh function that determines the fluid energy dissipation (mechanical-to-thermal energy conversion) according to the formula
  (10) $$\Phi =\frac{1}{2c_{p}}T:A_{1};$$
- the colon symbol: denotes the scalar product of tensors;
- $c_{p}$ is the heat capacity of the fluid, $c_{p}>0$;
- the operators div and ∇ are the divergence and the gradient, respectively, with respect to the space variables *x*, *y*, *z*;
- $\nabla^{2}\;\overset{\mathrm{def}}{=}\;\nabla \cdot \nabla =\frac{\partial^{2}}{\partial x^{2}}+\frac{\partial^{2}}{\partial y^{2}}+\frac{\partial^{2}}{\partial z^{2}}$.

**Figure 1:**
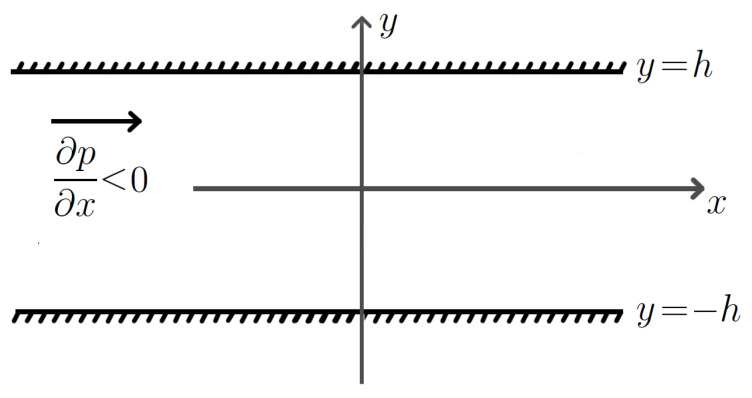
Flow configuration.

Let us consider the unidirectional fluid motion between horizontal parallel plates y=−h and y=h, assuming that the flow is driven by a constant pressure gradient

(11) $$\frac{\partial p}{\partial x}=-\xi ,\;\xi =\mathrm{const},\;\xi >0,$$

and

(12) $$g={\left(0,-g,0\right)}^{⊤},\;\theta =\theta \left(y\right),\;\omega =0,$$

where *g* is the value of acceleration due to gravity. This means that we deal with the *plane Poiseuille flow*. Figure 1 shows the chosen coordinate system and the flow geometry.

For such flow, we obviously have

$$v_{1}=u,\;v_{2}=0,\;v_{3}=0,$$

where u=u(y) is an unknown function. Then the following equalities hold:

(13) ∇·v=0,(v·∇)v=0,(v·∇)θ=0.

In view of relations (12) and (13), system (7)–(9) reduces to

(14) $$\begin{array}{c} \mathrm{div}T+\rho g=0, \end{array}$$

(15) $$\begin{array}{c} -\kappa \theta^{\prime \prime }=\Phi , \end{array}$$

where the symbol ${}^{\prime }$ denotes the differentiation with respect to *y*.

Assuming that the fluid obeys the constitutive relation (6), we rewrite (14) in the form

(16) $$\mathrm{div}(\mu A_{1}+\alpha A_{2}-\alpha A_{1}^{2})=\nabla p-\rho g.$$

We will use the nonlinear system (15), (16) for handling second grade fluid flows in the channel −h≤y≤h. Note that the unknowns of this system are *u*, *p*, and θ, while all other quantities are assumed to be given.

Of course, in order to obtain physically important solutions, Equations (15) and (16) must be supplemented with appropriate boundary conditions for the velocity field and the temperature. Experimental data and theoretical works point to different possibilities for the behaviour of fluid flows on solid walls. Along with the standard no-slip condition, various slip conditions are widely used (see, e.g., [23], § 5).

In this paper, we will investigate four boundary value problems describing flows of second grade fluids in the plane channel with impermeable solid walls.

**Problem 1.**  *Find a triplet (u,p,θ) that satisfies system *(15)*, *(16)* supplemented with the no-slip boundary condition*

(17) v(±h)=0

*and the Robin boundary condition for the temperature θ*

(18) $$\kappa \theta^{\prime }\left(\pm h\right)=\mp \beta \theta \left(\pm h\right),$$

*where β is a positive coefficient that characterizes the heat transfer on the channel walls.*

**Problem 2.**  *Find a triplet (u,p,θ) that satisfies system *(15)*, *(16)* supplemented with the threshold slip conditions on the plates y=±h:*

(19) $$\begin{array}{cc} \; & v\cdot n=0, \end{array}$$

(20) $$\begin{array}{cc} \; & {∥\left(Tn\right)}_{\tan}{∥}_{R^{3}}\leq \sigma \;⟹\;v_{\tan}=0, \end{array}$$

(21) $$\begin{array}{cc} \; & {∥\left(Tn\right)}_{\tan}{∥}_{R^{3}}>\sigma \;⟹\;{\left(Tn\right)}_{\tan}=-(\sigma +k∥v_{\tan}{∥}_{R^{3}})\frac{v_{\tan}}{∥v_{\tan}{∥}_{R^{3}}} \end{array}$$

*and boundary condition *(18)* for the temperature θ.*

Here, and in the succeeding discussion, the following notations are used:

- n is the exterior unit normal vector on the channel walls;
- $v_{\tan}$ denotes the tangential component of v;
- *k* is the slip coefficient, k>0;
- σ is the threshold value of the tangential stresses, σ≥0.

Equality (19) represents the impermeability condition on the channel walls. Relations (20) and (21) mean that the fluid slips at a point on the boundary if and only if the magnitude of the tangential traction exceeds the slip threshold σ. These conditions are called the *threshold slip conditions* as well as the *Navier–Fujita slip conditions* [51].

**Problem 3.**  *Find a triplet (u,p,θ) that satisfies system *(15)*, *(16)* under the mixed conditions for the velocity field v and the temperature θ:*

$$\begin{array}{cc} \; & v\cdot n=0\;\mathrm{on}\;\mathrm{the}\;\mathrm{plate}\;y=h, \end{array}$$

(22) $$\begin{array}{cc} \; & {∥\left(Tn\right)}_{\tan}{∥}_{R^{3}}\leq \sigma \;⟹\;v_{\tan}=0\;\mathrm{on}\;\mathrm{the}\;\mathrm{plate}\;y=h, \end{array}$$

(23) $$\begin{array}{cc} \; & {∥\left(Tn\right)}_{\tan}{∥}_{R^{3}}>\sigma \;⟹\;{\left(Tn\right)}_{\tan}=-(\sigma +k∥v_{\tan}{∥}_{R^{3}})\frac{v_{\tan}}{∥v_{\tan}{∥}_{R^{3}}}\;\mathrm{on}\;\mathrm{the}\;\mathrm{plate}\;y=h, \end{array}$$

(24) $$\begin{array}{cc} \; & v=0\;\mathrm{on}\;\mathrm{the}\;\mathrm{plate}\;y=-h, \end{array}$$

(25) $$\begin{array}{cc} \; & \theta^{\prime }=0\;\mathrm{on}\;\mathrm{the}\;\mathrm{plate}\;y=-h, \end{array}$$

(26) $$\begin{array}{cc} \; & \theta =0\;\mathrm{on}\;\mathrm{the}\;\mathrm{plate}\;y=h. \end{array}$$

**Problem 4.**  *Find a triplet (u,p,θ) that satisfies system *(15)*, *(16)* under the mixed conditions for the velocity field v and the temperature θ:*

(27) $$\begin{array}{cc} \; & v\cdot n=0\;\mathrm{on}\;\mathrm{the}\;\mathrm{plates}\;y=\pm h, \end{array}$$

(28) $$\begin{array}{cc} \; & {∥\left(Tn\right)}_{\tan}{∥}_{R^{3}}\leq \sigma \;⟹\;v_{\tan}=0\;\mathrm{on}\;\mathrm{the}\;\mathrm{plate}\;y=h, \end{array}$$

(29) $$\begin{array}{cc} \; & {∥\left(Tn\right)}_{\tan}{∥}_{R^{3}}>\sigma \;⟹\;{\left(Tn\right)}_{\tan}=-(\sigma +k_{1}∥v_{\tan}{∥}_{R^{3}})\frac{v_{\tan}}{∥v_{\tan}{∥}_{R^{3}}}\;\mathrm{on}\;\mathrm{the}\;\mathrm{plate}\;y=h, \end{array}$$

(30) $$\begin{array}{cc} \; & {\left(Tn\right)}_{\tan}=-k_{2}v_{\tan}\;\mathrm{on}\;\mathrm{the}\;\mathrm{plate}\;y=-h, \end{array}$$

(31) $$\begin{array}{cc} \; & \theta =0\;\mathrm{on}\;\mathrm{the}\;\mathrm{plates}\;y=\pm h, \end{array}$$

*where $k_{1}>0$ and $k_{2}\geq 0$.*

Note that condition (30) states that the fluid slips on the solid wall for any non-zero shear stresses. This situation corresponds to the limit case as $\sigma \to 0^{+}$ for the threshold slip conditions. In the literature, equality (30) is referred to as the *Navier slip condition*, after Navier [52] who first proposed it. The corresponding slip regime is sometimes referred to as the *free slip* [23]. However, this condition should not be confused with the *perfect slip condition* ${\left(Tn\right)}_{\tan}=0$ (see [53,54,55]), which is valid only when $k_{2}=0$. As noted in [37], Navier’s slip condition can be considered as a homotopy transformation that links the no-slip boundary condition on the one hand with the no-stick boundary condition on the other hand.

## 3. Analysis and Exact Solution of Problem 1

First we calculate the Rivlin–Ericksen tensors $A_{1}$ and $A_{2}$:

(32) $$A_{1}=\left[\begin{array}{ccc} 0\; & u^{\prime }\left(y\right)\; & 0\\ u^{\prime }\left(y\right)\; & 0\; & 0\\ 0\; & 0\; & 0 \end{array}\right],\;A_{2}=\left[\begin{array}{ccc} 0\; & 0\; & 0\\ 0\; & 2{\left(u^{\prime }\left(y\right)\right)}^{2}\; & 0\\ 0\; & 0\; & 0 \end{array}\right].$$

Next, using these equalities and (6), we obtain

$$A_{1}^{2}=\left[\begin{array}{ccc} {\left(u^{\prime }\left(y\right)\right)}^{2}\; & 0\; & 0\\ 0\; & {\left(u^{\prime }\left(y\right)\right)}^{2}\; & 0\\ 0\; & 0\; & 0 \end{array}\right],$$

(33) $$T=-p\left(x,y,z\right)I+\left[\begin{array}{ccc} -\alpha {\left(u^{\prime }\left(y\right)\right)}^{2}\; & \mu u^{\prime }\left(y\right)\; & 0\\ \mu u^{\prime }\left(y\right)\; & \alpha {\left(u^{\prime }\left(y\right)\right)}^{2}\; & 0\\ 0\; & 0\; & 0 \end{array}\right]$$

and rewrite (16) in the form

$$\mathrm{div}\left[\begin{array}{ccc} -\alpha {\left(u^{\prime }\left(y\right)\right)}^{2}\; & \mu u^{\prime }\left(y\right)\; & 0\\ \mu u^{\prime }\left(y\right)\; & \alpha {\left(u^{\prime }\left(y\right)\right)}^{2}\; & 0\\ 0\; & 0\; & 0 \end{array}\right]=\nabla p\left(x,y,z\right)-\rho g.$$

The last equation is equivalent to the following system:

(34) $$\begin{array}{cc} \mu u^{\prime \prime }\left(y\right) & =\frac{\partial p(x,y,z)}{\partial x}, \end{array}$$

(35) $$\begin{array}{cc} \alpha {\left[{\left(u^{\prime }\left(y\right)\right)}^{2}\right]}^{\prime } & =\frac{\partial p(x,y,z)}{\partial y}+\rho g, \end{array}$$

(36) $$\begin{array}{cc} 0 & =\frac{\partial p(x,y,z)}{\partial z}. \end{array}$$

From (10) and the first equality of (32) it follows that

$$\Phi =\frac{\mu }{c_{p}}{\left(u^{\prime }\left(y\right)\right)}^{2}.$$

Using this equality, we rewrite (15) as follows:

(37) $$-\kappa \theta^{\prime \prime }\left(y\right)=\frac{\mu }{c_{p}}{\left(u^{\prime }\left(y\right)\right)}^{2}.$$

Note that system (34)–(37) can be considered as a starting point for solving all the boundary value problems that are stated in this paper.

Let us construct the exact solution to Problem 1. First, we will find the pressure *p*. In view of (36), the pressure is independent of *z*, that is, p=p(x,y). Moreover, taking into account condition (11), we conclude that the pressure should be sought in the form

p(x,y)=−ξx+ϕ(y)

with an unknown function ϕ=ϕ(y).

From (35) it follows that

$$\phi \left(y\right)=\alpha {\left(u^{\prime }\left(y\right)\right)}^{2}-\rho gy+C,$$

where *C* is a constant. By setting C=ρgh, we obtain

(38) $$\phi \left(y\right)=\alpha {\left(u^{\prime }\left(y\right)\right)}^{2}+\rho g\left(h-y\right).$$

From (11) and (34) it follows that

(39) $$\mu u^{\prime }\left(y\right)=-\xi y+C_{0},$$

where $C_{0}$ is a constant.

In view of the physical meaning of Problem 1, the velocity field is symmetric with respect to the plane y=0, that is, the function u=u(y) is even. Hence, we have $u^{\prime }\left(0\right)=0$. Setting y=0 in (39), we obtain $C_{0}=0$. Therefore,

(40) $$u^{\prime }\left(y\right)=-\frac{\xi }{\mu }y.$$

Substituting (40) into (38), we arrive at

$$\phi \left(y\right)=\frac{\alpha \xi^{2}}{\mu^{2}}y^{2}+\rho g\left(h-y\right).$$

Next, we shall find *u*. From (40) it follows that

(41) $$u\left(y\right)=-\frac{\xi }{2\mu }y^{2}+C_{1}.$$

It is clear that the value of the constant $C_{1}$ must be chosen such that the no-slip condition (17) on the channel walls is satisfied. Since *u* is an even function, it suffices to verify that the boundary condition holds on the upper wall. Setting y=h in (41), we find $C_{1}=\xi h^{2}/\left(2\mu \right)$.

We now know the function *u* and hence, in order to find the temperature distribution in the channel, we can solve the boundary value problem (18), (37) with respect to θ.

Thus, we have obtained the exact solution to Problem 1:

(42) $$\begin{array}{cc} u(y)\; & =-\frac{\xi }{2\mu }\left(y^{2}-h^{2}\right),\\ p(x,y) & =-\xi x+\frac{\alpha \xi^{2}}{\mu^{2}}y^{2}+\rho g\left(h-y\right),\\ \theta (y)\; & =-\frac{\xi^{2}}{12c_{p}\mu \kappa }y^{4}+\frac{\xi^{2}h^{3}\left(h\beta +4\kappa \right)}{12c_{p}\mu \beta \kappa }. \end{array}$$

## 4. Analysis and Exact Solution of Problem 2

Now let us consider the case of the threshold slip boundary conditions (19)–(21).

With the help of arguments similar to those given in the previous section, we can verify the validity of relations (38), (40), and (41) for Problem 2.

Next, directly calculating

$$\begin{array}{cc} {Tn|}_{y=\pm h}= & -p\left(x,\pm h\right)\left[\begin{array}{ccc} 1 & 0 & 0\\ 0 & 1 & 0\\ 0 & 0 & 1 \end{array}\right]\left(\begin{array}{c} 0\\ \pm 1\\ 0 \end{array}\right)\\ \; & +\left[\begin{array}{ccc} -\alpha {\left(u^{\prime }\left(\pm h\right)\right)}^{2} & \mu u^{\prime }\left(\pm h\right) & 0\\ \mu u^{\prime }\left(\pm h\right) & \alpha {\left(u^{\prime }\left(\pm h\right)\right)}^{2} & 0\\ 0 & 0 & 0 \end{array}\right]\left(\begin{array}{c} 0\\ \pm 1\\ 0 \end{array}\right)\\ = & \left(\begin{array}{c} \pm \mu u^{\prime }\left(\pm h\right)\\ \mp p\left(x,\pm h\right)\pm \alpha {\left(u^{\prime }\left(\pm h\right)\right)}^{2}\\ 0 \end{array}\right), \end{array}$$

and using (40), we derive

$$∥{\left(Tn{|}_{y=\pm h}\right)}_{\tan}{∥}_{R^{3}}=\left|\mu u^{\prime }\left(\pm h\right)\right|=\xi h.$$

Taking into account the last relation and (19)–(21), we separately consider two cases: ξh≤σ and ξh>σ.

If the inequality ξh≤σ is valid, then in view of equality (20), there is no boundary slip and the velocity field in the channel is determined by formula (42) as in Problem 1.

If the inequality ξh>σ holds, then the fluid velocity on the channel walls is non-zero. In view of (21), the following equalities hold:

$$\pm \mu u^{\prime }\left(\pm h\right)=-\left(\sigma +ku\left(\pm h\right)\right).$$

Taking into account (40), we obtain

−ξh=−(σ+ku(±h)),

and hence

$$u\left(\pm h\right)=\frac{\xi h-\sigma }{k}.$$

Using this equality and (41), we find the function *u*:

$$u\left(y\right)=-\frac{\xi }{2\mu }\left(y^{2}-h^{2}\right)+\frac{\xi h-\sigma }{k}.$$

In both cases, to obtain the temperature distribution in the channel, it is sufficient to solve equation (37) under boundary condition (18) with respect to the function θ.

Combining the solutions constructed for each of the above-mentioned cases, we obtain the general solution to Problem 2, which satisfies all imposed conditions:

$$\begin{array}{cc} u(y)\; & =-\frac{\xi }{2\mu }\left(y^{2}-h^{2}\right)+\frac{\left(1-H(\sigma -\xi h))(\xi h-\sigma \right)}{k},\\ p(x,y) & =-\xi x+\frac{\alpha \xi^{2}}{\mu^{2}}y^{2}+\rho g\left(h-y\right),\\ \theta (y)\; & =-\frac{\xi^{2}}{12c_{p}\mu \kappa }y^{4}+\frac{\xi^{2}h^{3}\left(h\beta +4\kappa \right)}{12c_{p}\mu \beta \kappa }, \end{array}$$

where *H* is the Heaviside step function defined by

$$H\left(s\right)\overset{\mathrm{def}}{=}\left\{\begin{array}{c} 0\;\;\mathrm{if}s<0,\\ 1\;\;\mathrm{if}s\geq 0. \end{array}\right.$$

## 5. Analysis and Exact Solution of Problem 3

For flow models with mixed boundary conditions on the channel walls, one must keep in mind that the velocity field is not symmetric with respect to the plane y=0, and hence relation (40) may not hold. Therefore, we turn to relation (34), from which, after integrating with respect to *y*, we find

(43) $$\mu u\left(y\right)=-\frac{\xi }{2}y^{2}+C_{1}y+C_{2},$$

where $C_{1}$ and $C_{2}$ are some constants.

Using boundary condition (24), we derive from (43) that

$$C_{2}=\frac{\xi }{2}h^{2}+C_{1}h.$$

Substituting $C_{2}$ into (43), we arrive at the relation

(44) $$\mu u\left(y\right)=-\frac{\xi }{2}\left(y^{2}-h^{2}\right)+C_{1}\left(y+h\right).$$

Now let us find the value of the constant $C_{1}$ based on the threshold slip condition on the upper wall of the channel. Differentiating both sides of identity (43) with respect to *y*, we obtain

(45) $$\mu u^{\prime }\left(y\right)=-\xi y+C_{1}.$$

Taking into account (33) and (45), we derive

(46) $${\left(Tn{|}_{y=h}\right)}_{\tan}=\left(\begin{array}{c} \mu u^{\prime }\left(h\right)\\ 0\\ 0 \end{array}\right)=\left(\begin{array}{c} -\xi h+C_{1}\\ 0\\ 0 \end{array}\right).$$

Let us consider separately the two cases: the no-slip regime and the slip regime on the wall y=h.

If the fluid adheres to the wall y=h (mathematically, this means that u(h)=0), then from (44) it follows that $C_{1}=0$. In view of condition (22), this regime is realized if

$$∥{\left(Tn{|}_{y=h}\right)}_{\tan}{∥}_{R^{3}}=\xi h\leq \sigma .$$

The slip regime arises if

$$∥{\left(Tn{|}_{y=h}\right)}_{\tan}{∥}_{R^{3}}=\left|-\xi h+C_{1}\right|>\sigma .$$

In view of condition (23), the following equality holds:

$$\mu u^{\prime }\left(h\right)=-\left(\sigma +k|u\left(h\right)|\right)\;sgn\left(u\left(h\right)\right).$$

Using (44) and (45), we rewrite the last equality as follows:

(47) $$-\xi h+C_{1}=-\left(\sigma +\frac{2kh|C_{1}|}{\mu }\right)sgn\left(C_{1}\right).$$

This implies, in particular, that $C_{1}>0$. Therefore, (47) reduces to

$$-\xi h+C_{1}=-\left(\sigma +\frac{2khC_{1}}{\mu }\right),$$

where $C_{1}=\mu (\xi h-\sigma )/\left(\mu +2hk\right)$. Combining this with $C_{1}>0$, we arrive at the inequality ξh>σ indicating the slip regime.

After finding the velocity component *u*, one can derive the temperature θ from system (15), (25), and (26).

Thus, we have obtained the general solution of Problem 3, which is suitable for any admissible values of the model parameters:

$$\begin{array}{cc} u(y)\;= & -\frac{\xi }{2\mu }\left(y^{2}-h^{2}\right)+\psi \left(y+h\right),\\ p(x,y)= & -\xi x+\frac{\alpha }{\mu^{2}}{\left(\xi y-\psi \mu \right)}^{2}+\rho g\left(h-y\right),\\ \theta (y)\;= & -\frac{{\left(\xi y-\psi \mu \right)}^{4}}{12c_{p}\mu \kappa \xi^{2}}-\frac{{\left(\psi \mu +h\xi \right)}^{3}}{3c_{p}\mu \kappa \xi }y+\frac{\psi^{4}\mu^{4}+18\psi^{2}h^{2}\mu^{2}\xi^{2}+8\psi h^{3}\mu \xi^{3}+5h^{4}\xi^{4}}{12c_{p}\mu \kappa \xi^{2}}, \end{array}$$

where

$$\psi \overset{\mathrm{def}}{=}\frac{\left(1-H(\sigma -\xi h))(\xi h-\sigma \right)}{\mu +2hk}.$$

## 6. Analysis and Exact Solution of Problem 4

Obviously, for solving Problem 4 one can use relations (43), (45), and (46). Two cases are possible: either the no-slip condition holds on the plate y=h, or the slip regime is realized on this plate.

First, let us find the solution for the first case. Substitute y=h into (43). Since u(h)=0, we see that

$$C_{2}=\frac{\xi h^{2}}{2}-C_{1}h,$$

and hence

(48) $$u\left(y\right)=-\frac{\xi }{2\mu }\left(y^{2}-h^{2}\right)+\frac{C_{1}}{\mu }\left(y-h\right).$$

Further, let us choose the value of the constant $C_{1}$ such that the Navier slip condition

(49) $$-\mu u^{\prime }\left(-h\right)=-k_{2}u\left(-h\right)$$

is satisfied on the lower wall of the channel. Taking into account (45) and (48), we rewrite (49) as follows

$$-\xi h-C_{1}=\frac{2hk_{2}C_{1}}{\mu },$$

where

(50) $$C_{1}=-\frac{\xi h\mu }{\mu +2k_{2}h}.$$

Let us now determine relations on the model parameters under which the above case is realized. In view of (28), the following inequality

(51) $${∥\left(Tn\right)}_{\tan}{∥}_{R^{3}}\leq \sigma$$

holds for y=h. Using (46) and (50), we conclude that (51) is true if

(52) $$\xi h\leq \tilde{\sigma }\overset{\mathrm{def}}{=}\sigma \left(1-\frac{\mu }{2(\mu +k_{2}h)}\right).$$

Let us now consider the case when the slip regime is realized on the upper wall of the channel. Then, boundary conditions (27)–(30) reduce to the following system:

(53) $$\begin{array}{cc} \mu u^{\prime }\left(h\right)\;\;\; & =-(\sigma +k_{1}u\left(h\right)), \end{array}$$

(54) $$\begin{array}{cc} -\mu u^{\prime }\left(-h\right) & =-k_{2}u\left(-h\right). \end{array}$$

Using (43) and (45), one can rewrite (53) and (54) as follows:

$$\begin{array}{cc} -\xi h+C_{1}\;\;\; & =-\left(\sigma +k_{1}\left(-\frac{\xi h^{2}}{2\mu }+\frac{C_{1}h}{\mu }+\frac{C_{2}}{\mu }\right)\right),\\ -(\xi h+C_{1}) & =-k_{2}\left(-\frac{\xi h^{2}}{2\mu }-\frac{C_{1}h}{\mu }+\frac{C_{2}}{\mu }\right). \end{array}$$

Solving this system, we find the values of the constants $C_{1}$ and $C_{2}$:

C1=|−(ξh(k1−k2)+σk2)μ2hk1k2+μ(k1+k2),C2=|2h3ξk1k2+3h2μξ(k1+k2)+4hμ2ξ−2hμσk2−2μ2σ4hk1k2+2μ(k1+k2).

Taking into account (29), it is easy to check that the case under consideration is realized if the following inequality holds:

(55) $$\xi h>\tilde{\sigma }.$$

When the function *u* is obtained, one can derive the temperature θ by solving (15) with the Dirichlet boundary condition (31).

Finally, summarizing the results of this section, we write the solution of Problem 4 in the explicit form: if the model parameters satisfy relation (52), then

$$\begin{array}{cc} u(y)\;= & -\frac{\xi }{2\mu }\left(y^{2}-h^{2}\right)+\lambda_{1}\left(y-h\right),\\ p(x,y)= & -\xi x+\frac{\alpha }{\mu^{2}}{\left(\xi y-\lambda_{1}\mu \right)}^{2}+\rho g\left(h-y\right),\\ \theta (y)\;= & -\frac{{\left(\xi y-\lambda_{1}\mu \right)}^{4}}{12c_{p}\mu \kappa \xi^{2}}-\frac{\lambda_{1}\left({\lambda_{1}}^{2}\mu^{2}+h^{2}\xi^{2}\right)}{3c_{p}\kappa \xi }y+\frac{{\lambda_{1}}^{4}\mu^{4}+6{\lambda_{1}}^{2}h^{2}\mu^{2}\xi^{2}+h^{4}\xi^{4}}{12c_{p}\mu \kappa \xi^{2}}, \end{array}$$

where

$$\lambda_{1}\overset{\mathrm{def}}{=}-\frac{\xi h}{\mu +2k_{2}h};$$

otherwise, when inequality (55) holds, the exact solution is determined by the following formulas:

$$\begin{array}{cc} u(y)\;= & -\frac{\xi }{2\mu }\left(y^{2}-h^{2}\right)+\lambda_{2}y+\frac{\xi h^{2}\left(k_{1}+k_{2}\right)+2\xi h\mu -\sigma hk_{2}-\mu \sigma }{2hk_{1}k_{2}+\mu \left(k_{1}+k_{2}\right)},\\ p(x,y)= & -\xi x+\frac{\alpha }{\mu^{2}}{\left(\xi y-\lambda_{2}\mu \right)}^{2}+\rho g\left(h-y\right),\\ \theta (y)\;= & -\frac{{\left(\xi y-\lambda_{2}\mu \right)}^{4}}{12c_{p}\mu \kappa \xi^{2}}-\frac{\lambda_{2}\left({\lambda_{2}}^{2}\mu^{2}+h^{2}\xi^{2}\right)}{3c_{p}\kappa \xi }y+\frac{{\lambda_{2}}^{4}\mu^{4}+6{\lambda_{2}}^{2}h^{2}\mu^{2}\xi^{2}+h^{4}\xi^{4}}{12c_{p}\mu \kappa \xi^{2}}, \end{array}$$

where

$$\lambda_{2}\overset{\mathrm{def}}{=}-\frac{\xi h\left(k_{1}-k_{2}\right)+\sigma k_{2}}{2hk_{1}k_{2}+\mu \left(k_{1}+k_{2}\right)}.$$

## 7. Conclusions

In this work, we have studied the non-isothermal steady-state flow of a second grade fluid in the channel −h≤y≤h with impermeable solid walls, taking into account the fluid energy dissipation (mechanical-to-thermal energy conversion) in the heat transfer equation. It is assumed that the flow is created by a constant pressure gradient ∂p/∂x=−ξ. We have established exact solutions of the nonlinear governing equations for the velocity vector, the pressure, and the temperature under the no-slip boundary conditions and threshold-type slip boundary conditions, which include Navier’s slip condition as a limit case. Moreover, we analytically solved two problems for channel flows with mixed boundary conditions, assuming that the upper and lower walls of the channel differ in their physical properties. The obtained solutions show that the pressure in the channel significantly depends on the normal stress coefficient α, especially in those layers where the change in the flow velocity in the transverse direction to the flow is large. At the same time, the velocity field is independent of α, and therefore coincides with the velocity field that occurs in the case of a Newtonian fluid (α=0). In the analysis of flows with threshold slip, the key point is the value of ξh. If ξh exceeds a given threshold value σ, then the slip regime holds at solid surfaces, otherwise the fluid adheres to the walls of the channel. If it is assumed that on one part of the boundary Navier’s condition is provided, while on the other one the threshold slip condition holds, then, for the slip regime, the associated threshold value $\tilde{\sigma }$ is reduced to a certain extent, but not more than twice. An interesting feature of the obtained results is that the temperature distribution is given by a fourth-degree polynomial, and not by a quadratic function. This is due to the fact that when deriving the heat transfer equation, the simplifying assumption that the viscous energy dissipation function is identically equal to zero is not used. The proposed approach leads to a more delicate description of the heat and mass transfer in second grade fluids as well as a deep understanding of the related physical processes. Finally, note that the exact solutions obtained in the present paper can be applied to testing numerical, asymptotic, and approximate analytical methods of solving boundary value problems that describe non-isothermal flows of nanofluids.

## Data Availability

Not applicable.
